# From Metabolism to Mind: The Cardio–Metabolic–Brain Axis and the Role of Insulin Resistance—A Review

**DOI:** 10.3390/biomedicines14020394

**Published:** 2026-02-09

**Authors:** Joanna Cielecka, Zuzanna Szkamruk, Maciej Walędziak, Anna Różańska-Walędziak

**Affiliations:** 1 Medical Faculty, Collegium Medicum, Cardinal Stefan Wyszynski University in Warsaw, 01-938 Warsaw, Poland; 127466@student.uksw.edu.pl (J.C.); 128287@student.uksw.edu.pl (Z.S.); 2 Department of General, Oncological, Metabolic and Thoracic Surgery, Military Institute of Medicine, 01-141 Warsaw, Poland; maciej.waledziak@gmail.com; 3 Department of Human Physiology and Pathophysiology, Faculty of Medicine, Collegium Medicum, Cardinal Stefan Wyszynski University in Warsaw, 01-938 Warsaw, Poland

**Keywords:** insulin resistance (IR), cardio–metabolic–brain axis, cardiovascular disease (CVD), cognitive decline, endothelial dysfunction, inflammation, neurodegeneration, Alzheimer’s disease, metabolic syndrome, obesity, type 2 diabetes mellitus

## Abstract

**(1) Background:** Insulin resistance (IR) is increasingly recognized not only as a key factor in metabolic and cardiovascular disorders but also as an important contributor to cognitive decline. The growing prevalence of obesity, type 2 diabetes mellitus, and cardiovascular disease (CVD), paralleled by rising rates of dementia, highlights the need for an integrative model linking these conditions. The emerging cardio–metabolic–brain axis proposes a unified model explaining how biomarkers of metabolic stress, adipose-tissue-derived mediators, and abnormalities in laboratory parameters interact with vascular injury and neurodegeneration. **(2) Methods:** A comprehensive literature review was conducted using MEDLINE, SCOPUS, and Web of Science databases, complemented by additional searches in Embase and Cochrane Library. Studies from the past decade were screened using keywords such as “insulin resistance”, “cardio-metabolic-brain axis”, “cognitive decline”, and “cardiovascular disease”. Both epidemiological and mechanistic studies were analyzed to summarize current evidence and identify research gaps. **(3) Results and Conclusions:** Evidence indicates that insulin resistance contributes to endothelial dysfunction, chronic inflammation, and oxidative stress, driving the metabolic abnormalities characteristic of obesity and type 2 diabetes and promoting both atherosclerosis and neurodegeneration. Individuals with elevated IR—regardless of diabetes status—display higher risks of cardiovascular events and measurable cognitive decline. Brain insulin resistance further impairs glucose utilization, disrupts synaptic function, and facilitates amyloid accumulation, reflecting mechanisms observed in Alzheimer’s disease. These findings support IR as a key biomarker linking metabolic stress, vascular injury, and neural vulnerability within the cardio–metabolic–brain axis. Early identification of IR, together with targeted lifestyle and pharmacological interventions, may therefore offer dual benefits for cardiovascular and brain health. Continued longitudinal research is needed to validate this integrative model and refine therapeutic strategies aimed at improving insulin sensitivity.

## 1. Introduction

### 1.1. Definition and Clinical Significance of the Topic

Insulin resistance (IR) represents a systemic disturbance in which target tissues such as skeletal muscle, liver, adipose tissue, and the brain exhibit a reduced biological response to insulin. This impaired insulin action disrupts glycemic homeostasis, leading to compensatory hyperinsulinemia as a physiological response aimed at maintaining normoglycemia. Initially considered a purely metabolic abnormality, IR is now recognized as a multisystem disorder that integrates metabolic, cardiovascular, and neural dysfunctions within a shared pathophysiological framework [1].

Epidemiological studies indicate that insulin resistance is highly prevalent in the general population, affecting a substantial proportion of individuals without diagnosed diabetes. IR is commonly observed in association with aging, obesity, visceral adiposity, and metabolic syndrome, but it may also be present in lean individuals and in early stages of metabolic dysregulation. Importantly, population-based cohorts have demonstrated that IR often precedes the development of type 2 diabetes and cardiovascular disease by several years, highlighting its role as an early and subclinical driver of cardiometabolic risk.

Accumulating evidence indicates that IR plays a central role in the pathogenesis of cardiometabolic diseases. By promoting endothelial dysfunction, oxidative stress, and chronic low-grade inflammation, IR accelerates atherosclerosis, contributes to arterial stiffness and hypertension, and increases the overall risk of cardiovascular disease even in the absence of overt diabetes. Consequently, IR is increasingly viewed as an independent and early marker of vascular pathology and a key determinant of cardiovascular health [2,3]. Beyond its peripheral metabolic effects, IR also influences the central nervous system. Insulin signaling in the brain is essential for neuronal metabolism, synaptic plasticity, and cognitive processing. When this signaling pathway becomes impaired, glucose utilization in neural tissue declines, neuroinflammatory responses intensify, and synaptic integrity is disrupted, contributing to cognitive dysfunction [4]. The convergence of these mechanisms has led to the formulation of the cardio–metabolic–brain axis—a conceptual model describing how metabolic dysregulation, vascular injury, and neural decline interact through shared molecular and physiological pathways [4,5]. These processes contribute to cognitive dysfunction and may facilitate the development of neurodegenerative diseases.

Emerging evidence suggests that so-called “brain insulin resistance” may occur independently of systemic glycemic status and is associated with impairments in memory, executive function, and learning, as well as with structural and functional brain alterations detectable by neuroimaging.

The convergence of these mechanisms has led to the formulation of the cardio–metabolic–brain axis—a conceptual model that describes how metabolic dysregulation, vascular injury, and neural decline interact through shared molecular and physiological pathways. In this context, the term “cardio–metabolic–brain axis” is used as a conceptual and integrative framework rather than a classical physiological axis. It does not imply hierarchical control by a single commander center, but instead reflects a network of bidirectional and maladaptive interactions among metabolic, vascular, and neural systems. The term “cardio” refers to the cardiovascular and vascular component mediating these interactions, rather than dominance of cardiac regulation. Within this framework, insulin resistance acts as a biological link connecting metabolic disorders, cardiovascular disease, and cognitive impairment [4]. Recognizing IR as a systemic rather than isolated metabolic condition carries significant clinical implications. It underscores the importance of early identification and multifaceted management aimed at improving insulin sensitivity to simultaneously protect metabolic balance, vascular integrity, and cognitive health [5].

Moreover, recent post-mortem and neuroimaging findings indicate that impaired insulin signaling in the brain is closely linked to late-life cognitive decline, even in individuals without diabetes, reinforcing the concept that brain insulin resistance represents a silent yet critical driver of neurodegenerative processes [6].

From a clinical and research perspective, insulin resistance can be assessed using a variety of laboratory-based and composite indices. While traditional measures such as HOMA-IR and fasting insulin remain widely used, recent years have seen the development of composite indices incorporating anthropometric and lipid parameters, including the triglyceride–glucose (TyG) index, TyG-derived indices (e.g., TyG-BMI, TyG-waist circumference), the estimated glucose disposal rate (eGDR), and the metabolic score for insulin resistance (METS-IR). These indices have demonstrated utility in large-scale epidemiological studies and in diverse patient populations, enabling improved detection of insulin resistance and its associated cardiometabolic and neurocognitive complications. [7,8,9]

Additionally, considering that IR can be detected through accessible laboratory biomarkers—including HOMA-IR, fasting insulin, lipid abnormalities, and inflammatory mediators—its identification may allow earlier recognition of individuals at heightened cardiometabolic and cognitive risk, providing an opportunity for preventive or targeted therapeutic intervention.

### 1.2. Purpose of the Study

The purpose of this study is to examine the integrative concept of the cardio–metabolic–brain axis and to evaluate the role of insulin resistance (IR) as a central mechanism linking metabolic, cardiovascular, and cognitive dysfunction. As the prevalence of insulin resistance, obesity, and type 2 diabetes continues to rise globally, parallel increases in cardiovascular disease and cognitive decline suggest a shared biological foundation. This review aims to synthesize current evidence describing how impaired insulin signaling contributes to vascular injury, metabolic imbalance, and neurodegeneration. By bringing together data from epidemiological, clinical, and mechanistic studies, the paper seeks to clarify the interconnected pathways through which IR affects both cardiovascular and brain health, and to highlight the need for early detection and targeted interventions that address this multidimensional risk.

## 2. Materials and Methods

A comprehensive literature review was conducted to explore the interconnections among insulin resistance (IR), cardiovascular disease (CVD), and cognitive decline within the emerging concept of the cardio–metabolic–brain axis. The primary objective of the search strategy was to identify studies examining epidemiological trends, biological mechanisms, and clinical outcomes that demonstrate how insulin resistance acts as a shared pathophysiological link between metabolic, vascular, and neurological systems.

The MEDLINE and SCOPUS scientific databases were searched systematically, and supplementary searches were performed in Embase, Cochrane Library, and Web of Science to ensure broad coverage of relevant international research. Boolean operators (AND, OR) were used to combine search terms, which included “insulin resistance”, “cardio-metabolic-brain axis”, “cardiovascular disease”, “cognitive decline”, “metabolic syndrome”, “endothelial dysfunction”, “neurodegeneration”, and “Alzheimer’s disease”. The search was limited to English-language articles published between January 2015 and December 2025. This time frame was selected to capture contemporary evidence reflecting current diagnostic criteria, advances in insulin resistance assessment, evolving neuroimaging techniques, and the recent development of integrative concepts such as brain insulin resistance and the cardio–metabolic–brain axis. Earlier studies were considered where necessary for historical context but were not systematically included in the primary search. Preference was given to original research papers, systematic reviews, and meta-analyses offering clinical, mechanistic, or epidemiological insight. Additional relevant papers were identified through manual screening of the reference lists of key studies. All databases were last consulted on 30 December 2025.

All retrieved titles and abstracts were independently screened by two reviewers according to predefined inclusion and exclusion criteria. Eligible studies included human research addressing the association between insulin resistance and either cardiovascular outcomes or cognitive function, as well as mechanistic studies describing biological pathways connecting these processes. Publications focusing solely on type 2 diabetes without reference to broader metabolic or neurological outcomes, case reports, non-peer-reviewed material, and studies lacking methodological clarity were excluded. Disagreements during study selection were resolved through discussion and consensus; when consensus could not be reached, a third independent reviewer was consulted. Data from each included article were extracted using a standardized form to ensure consistency in reporting of study design, participant characteristics, methods of insulin resistance assessment, cognitive or cardiovascular outcomes, and main findings.

Given the heterogeneity of study designs and outcome measures, a formal quantitative synthesis (meta-analysis) was not conducted. Instead, results were summarized narratively to provide a comprehensive qualitative overview of the current state of knowledge. A formal risk-of-bias evaluation using standardized tools such as the Newcastle–Ottawa Scale or ROBINS-I was not performed; however, the methodological quality of each study was assessed descriptively, taking into account design type, population size, confounder adjustment, and reporting completeness.

The review followed the Preferred Reporting Items for Systematic Reviews and Meta-Analyses (PRISMA 2020) guidelines to ensure methodological transparency and reproducibility. The protocol was not prospectively registered in PROSPERO or any other registry due to the exploratory nature of the project and time constraints. Nevertheless, all methodological steps—including database selection, Boolean search strings, and inclusion criteria—were documented to maintain reproducibility.

**Figure 1:**
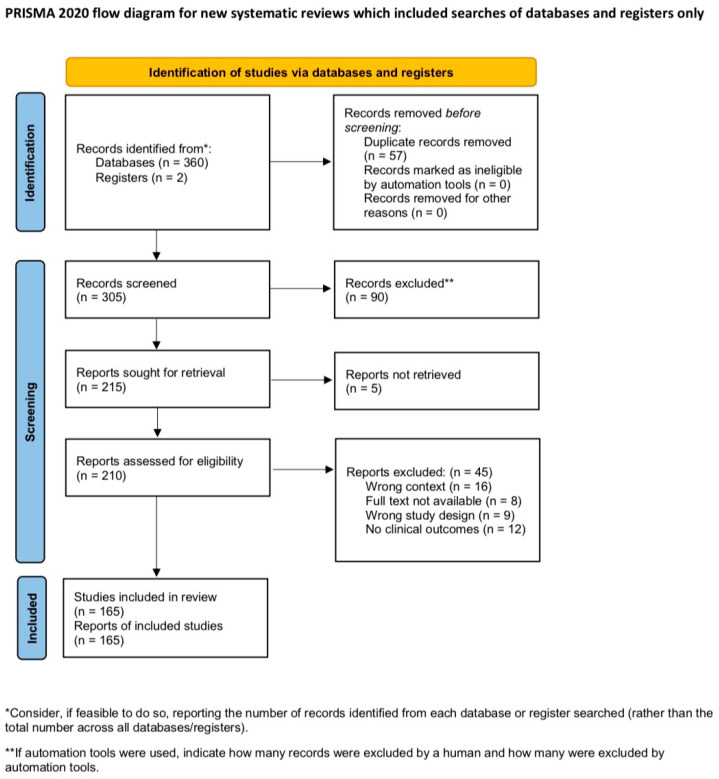
PRISMA 2020 flow diagram.

The selection process is illustrated in the PRISMA 2020 flow diagram (Figure 1), which outlines the number of records identified, screened, excluded, and ultimately included in the review. The structure of the analysis reflects the main thematic areas of current research: the epidemiological association between insulin resistance, cardiovascular disease, and cognitive dysfunction; underlying biological mechanisms forming the cardio–metabolic–brain axis; evidence from clinical and neuroimaging studies; and emerging preventive or therapeutic approaches targeting insulin sensitivity as a modifiable risk factor for both cardiovascular and brain health.

The included studies represented a range of study designs, including observational, cohort, and neuroimaging studies, and were conducted in diverse populations such as individuals from the general population and patients with obesity, insulin resistance, or type 2 diabetes, as well as older adults at increased cardiometabolic risk. The key characteristics and main findings of the included studies are summarized in a dedicated table to provide an overview of study design, population, and outcomes. In addition, the retrieved evidence is organized thematically in Section 3 according to specific subtopics reflecting the current state of the literature.

Summary of observational and population-based studies examining the association between insulin resistance, cardiovascular pathology, and cognitive or brain-related outcomes. Table 1 presents key characteristics of the included studies, including authors, publication year, journal, study design, and number of participants, together with the primary metabolic, cardiovascular, and neurocognitive outcomes assessed in each study. This overview highlights the heterogeneity of study populations and methodologies used to investigate the cardio–metabolic–brain network.

## 3. Results

### 3.1. Insulin Resistance—Definition, Assessment, and Clinical Significance

Insulin resistance (IR) is defined as a condition in which peripheral tissues such as skeletal muscle, liver, and adipose tissue exhibit a diminished ability to respond to circulating insulin, resulting in impaired glucose uptake and metabolic imbalance [10]. A key mechanism contributing to this defect is the decreased expression or translocation of the glucose transporter GLUT4 in insulin-responsive tissues, which represents a critical downstream step in insulin-stimulated glucose uptake, independent of the activity of upstream insulin signaling pathways. In addition to its peripheral manifestations, insulin signaling in the brain plays an essential role in maintaining neuronal metabolism, cognitive performance, and synaptic plasticity, and disturbances in this process have been described as brain insulin resistance. This neural form of IR has been associated with impaired glucose utilization in neurons, neuroinflammatory activation, and increased vulnerability to cognitive decline and Alzheimer’s disease.

The development of insulin resistance is closely linked to oxidative stress and endothelial dysfunction, both of which contribute to vascular inflammation and atherogenesis. These molecular disturbances not only impair vascular homeostasis but also create a chronic inflammatory environment that exacerbates cardiovascular and metabolic risk. Consequently, IR represents a critical pathophysiological link between metabolic dysregulation, vascular disease, and neurodegeneration [11,12,13].

Assessment of insulin resistance relies on both direct and indirect methods. The hyperinsulinemic–euglycemic clamp remains the gold standard for quantifying insulin sensitivity, but its complexity limits its use to research settings. Simpler surrogate indices, such as HOMA-IR and QUICKI, derived from fasting glucose and insulin levels, are widely applied in clinical and epidemiological studies due to their practicality and reliability [14,15]. Together, these approaches provide essential tools for identifying individuals at risk and for understanding how insulin resistance integrates metabolic, cardiovascular, and neurological dysfunction within a shared biological framework.

### 3.2. Overview of the Cardio–Metabolic–Brain Axis

Metabolic dysfunction—including insulin resistance, visceral adiposity, chronic low-grade inflammation, hyperglycemia, and dyslipidemia—constitutes a systemic pathophysiological environment that simultaneously affects multiple organ systems and integrates metabolic, vascular, cardiac, and neural injury into a single progressive axis. In this review, metabolic dysfunction is used as an umbrella term encompassing a spectrum of abnormalities, including insulin resistance, dyslipidemia, adipose tissue dysfunction, and chronic low-grade inflammation. Within this broader metabolic context, insulin resistance is emphasized as a central and mechanistically relevant component linking metabolic disturbances to cardiovascular pathology and brain dysfunction. At the cellular level, insulin resistance (IR) disrupts intracellular communication by selectively impairing the PI3K/Akt/eNOS signaling pathway, which normally stimulates nitric oxide (NO) synthesis and maintains endothelial quiescence. This impairment occurs in parallel with hyperactivation of the compensatory MAPK/ERK pathway, promoting endothelin-1 release, vasoconstriction, leukocyte adhesion, and increased vascular tone [16,17]. In visceral adipose tissue, hypertrophic adipocytes undergo hypoxic stress, activating HIF-1α and secreting elevated levels of TNF-α, IL-6, MCP-1, resistin, and free fatty acids (FFAs), while beneficial adipokines such as adiponectin and omentin decrease sharply. This imbalance contributes to a chronic meta-inflammatory state that exacerbates systemic IR and drives endothelial activation [18]. Macrophage infiltration into adipose depots, especially the shift from anti-inflammatory M2 to pro-inflammatory M1 phenotypes, further amplifies oxidative stress, ceramide generation, and cytokine release, linking metabolic injury to vascular inflammation.

Hyperglycemia deepens these disruptions through multiple biochemical mechanisms. Excess intracellular glucose overloads mitochondria, triggering excessive production of superoxide anions, formation of advanced glycation end products (AGEs), and persistent activation of the AGE-RAGE signaling cascade. These processes sustain NF-κB activation, promote the expression of pro-inflammatory cytokines, upregulate adhesion molecules (ICAM-1, VCAM-1, E-selectin), and induce eNOS uncoupling, reducing NO availability while increasing peroxynitrite formation. In parallel, atherogenic dyslipidemia—characterized by elevated triglyceride-rich lipoproteins, decreased HDL functionality, and enrichment of small dense LDL particles—facilitates transendothelial lipid transport, retention of ApoB-containing particles in the intima, and formation of oxidized LDL. The oxidized lipids stimulate foam-cell formation, smooth-muscle proliferation, and destabilization of the fibrous cap, thereby accelerating both early and advanced stages of atherosclerosis [19,20]. Together, these metabolic stressors generate vascular rarefaction, increased arterial stiffness, microvascular dropout, and loss of endothelial regenerative capacity, setting the stage for system-wide ischemia and tissue vulnerability.

These vascular changes have profound consequences for the heart. Cardiomyocytes, normally highly metabolic and dependent on tightly regulated mitochondrial function, are particularly vulnerable to the metabolic shifts imposed by insulin resistance and chronic lipid overload. In IR states, impaired glucose uptake forces the myocardium to rely excessively on fatty-acid β-oxidation, which is less oxygen-efficient and increases mitochondrial ROS production. Accumulation of lipid intermediates such as ceramides and diacylglycerols disrupts mitochondrial membrane integrity, induces mitochondrial fission, activates apoptotic pathways (caspase-3, caspase-9), and interferes with insulin signaling via PKCθ activation [21,22]. The resulting mitochondrial dysfunction contributes to reduced ATP availability, impaired sarcoplasmic reticulum Ca^2+^ cycling (via SERCA2a inhibition and RyR2 leakage), decreased contractile efficiency, and progressive diastolic dysfunction.

The impact of metabolic dysfunction on the myocardium is further compounded by glycation products, particularly methylglyoxal, which modify key proteins involved in excitation–contraction coupling and promote stiffening of cardiac tissues through collagen cross-linking. Experimental evidence demonstrates that methylglyoxal directly disrupts intracellular calcium handling, depresses myofilament sensitivity to Ca^2+^, and promotes arrhythmogenic activity, contributing to the development of diabetic cardiomyopathy even in the absence of overt coronary artery disease [23,24]. Meanwhile, microvascular dysfunction reduces coronary flow reserve, promotes myocardial ischemia, and heightens vulnerability to fibrotic remodeling. Clinically, large cardiovascular outcome trials demonstrate that modifying upstream metabolic abnormalities—using GLP-1 receptor agonists to reduce inflammation and weight, or SGLT2 inhibitors to enhance cardiac energetics and reduce renal–cardiac stress—significantly lowers cardiovascular mortality and major adverse cardiac events, validating the central role of metabolic drivers in cardiac disease [25,26,27].

In the brain, metabolic dysfunction triggers a cascade of microvascular and neuroinflammatory processes that progressively impair neural function. Hyperglycemia, dyslipidemia, and IR impair neurovascular coupling—the mechanism that matches neuronal activity with blood flow—by damaging endothelial cells, pericytes, and astrocytic endfeet that form the neurovascular unit (NVU). Structural changes such as thickening of the basement membrane, loss of capillary density, and endothelial tight-junction disruption weaken the blood–brain barrier (BBB), enabling entry of peripheral cytokines, lipoproteins, and albumin into the brain parenchyma. These intrusions activate microglia and astrocytes, shifting them toward pro-inflammatory phenotypes (M1-like microglia and A1 astrocytes) that release TNF-α, IL-1β, and complement proteins, accelerating synaptic loss and white-matter degeneration [28,29].

Disturbances in lipid metabolism further exacerbate neural vulnerability. Cholesterol and phospholipid homeostasis are essential for synaptic turnover, myelin maintenance, and signal transduction. In states of dyslipidemia and IR, glial cells—particularly astrocytes and oligodendrocytes—lose their capacity to maintain proper lipid recycling, impairing myelin sheath integrity and reducing neuronal resilience [30,31,32]. These molecular phenomena manifest clinically as impaired cerebral blood flow; hypoxia; reduced metabolic flexibility; and progressive small-vessel disease features such as white-matter hyperintensities, microbleeds, lacunes, and cortical thinning. Neuroimaging studies confirm strong associations between metabolic markers (HbA1c, insulin levels, HOMA-IR, TG/HDL ratio) and decreased white-matter microstructural integrity, diffuse hypoperfusion, and cognitive impairment, supporting the concept that metabolic dysfunction accelerates brain aging [33,34].

Collectively, these mechanisms form an integrative cardio–metabolic–brain framework, illustrating how metabolic injury initiates and sustains maladaptive interactions between vascular and neural systems, ultimately contributing to multisystem disease. Endothelial dysfunction integrates metabolic stress with macrovascular and microvascular pathology, linking dysregulated adipose tissue, mitochondria, and glucose/lipid metabolism to cardiovascular and neurological sequelae. Through the circulation, these vascular insults propagate from the periphery to the heart and brain, influencing cellular signaling, tissue energetics, inflammatory tone, and organ-level physiology. This interconnected network of metabolic, vascular, and neural interactions explains why insulin resistance, obesity, and dyslipidemia increase the risk not only of atherosclerosis and heart failure, but also of cerebral microangiopathy, vascular cognitive impairment, and increased susceptibility to neurodegenerative disease [35,36]. Recognizing this shared metabolic–vascular–cardio–neuro axis underscores the need for integrated therapeutic strategies targeting metabolism early in disease progression in order to preserve vascular health, optimize cardiac performance, and protect long-term cognitive and neural integrity.

### 3.3. Pathophysiological Role of Insulin Resistance in Cardiovascular Disease

Insulin resistance (IR) constitutes a unifying metabolic disturbance that promotes cardiovascular disease (CVD) through convergent and mutually reinforcing biological pathways. At the endothelial level, impaired insulin signalling diminishes endothelial nitric oxide synthase (eNOS) activation and nitric oxide (NO) bioavailability, producing endothelial dysfunction characterized by impaired vasodilatation, increased vascular tone and a pro-thrombotic surface. Compensatory hyperinsulinemia further shifts vascular homeostasis by stimulating vascular smooth muscle cell proliferation, extracellular matrix deposition and vascular remodeling, thereby increasing arterial stiffness and lowering the threshold for atheroma formation. These vascular consequences of IR are described in mechanistic reviews that link defects in insulin receptor substrate signalling to diminished NO, upregulation of endothelin-1 and enhanced vasoconstrictive responses [37,38].

Metabolic derangements that accompany IR directly accelerate atherogenesis. Insulin-resistant states foster an atherogenic lipoprotein profile—elevated triglycerides, increased hepatic very-low-density lipoprotein (VLDL) secretion, accumulation of small, dense LDL particles and low HDL cholesterol—which together increase arterial lipid infiltration and retention. At the same time, altered adipose tissue biology in IR (adipocyte hypertrophy, macrophage infiltration) leads to chronic low-grade inflammation with elevated circulating cytokines (TNF-α, IL-6) and adipokine dysregulation; these inflammatory mediators promote endothelial activation, leukocyte recruitment, matrix metalloproteinase expression and fibrous cap weakening. Oxidative stress—from mitochondrial dysfunction and NADPH oxidase activation—amplifies these processes by oxidizing lipoproteins and impairing vasoprotective signalling, thereby increasing plaque vulnerability. Recent comprehensive reviews synthesize these lipid–inflammatory–oxidative links and their role in plaque initiation, progression and destabilization [35,39].

IR also couples to hemodynamic and renal mechanisms that raise blood pressure and cardiovascular strain. Hyperinsulinemia promotes renal sodium reabsorption and augments sympathetic nervous system activity; IR-associated activation of the renin–angiotensin–aldosterone system (RAAS) promotes vasoconstriction, vascular hypertrophy and sodium retention, producing or worsening hypertension—a potent, independent driver of CVD. In addition, mTOR and related nutrient-sensing pathways implicated in IR influence vascular and myocardial hypertrophic signalling, linking metabolic excess to structural cardiovascular remodeling. These mechanistic linkages explain why IR frequently co-exists with hypertension and why combined metabolic–hemodynamic burdens accelerate end-organ damage [37,38].

Direct myocardial effects of IR further increase cardiovascular risk beyond atherosclerosis. In cardiomyocytes, insulin resistance alters substrate utilization (reduced glucose uptake, increased reliance on fatty acid oxidation), promoting lipid accumulation, lipotoxic intermediates, mitochondrial dysfunction and impaired energetics. Over time these metabolic insults contribute to maladaptive cardiac remodeling, impaired diastolic function and a phenotype prone to heart failure with preserved ejection fraction (HFpEF). Clinical cohorts and mechanistic reviews have reported associations between higher IR surrogates and worse outcomes in heart failure populations, and experimental data show that improving insulin sensitivity can ameliorate myocardial inflammation and fibrosis in preclinical models [40,41].

Imaging and biomarker studies provide translational evidence that systemic IR maps onto local vascular inflammation and plaque biology. Non-invasive markers such as pericoronary adipose tissue attenuation (on coronary CT) have been correlated with IR indices (e.g., TyG), demonstrating greater perivascular inflammation in insulin-resistant patients with coronary artery disease. Such imaging data bridge the mechanistic concept of IR-driven vascular inflammation with measurable anatomic risk (vulnerable plaques, perivascular inflammatory signatures), strengthening the biological plausibility of epidemiologic associations [42].

Epidemiologic evidence from 2023–2025 robustly supports IR as an independent predictor of cardiovascular events across diverse populations and using multiple IR surrogates. Multiple large meta-analyses and pooled cohort investigations have reported that higher values of widely used indices—including the triglyceride–glucose (TyG) index, the Metabolic Score for Insulin Resistance (METS-IR), estimated glucose disposal rate (eGDR), HOMA-IR and composite TyG-BMI metrics—are associated with a substantially increased risk of major adverse cardiovascular events (MACEs), myocardial infarction, stroke and cardiovascular mortality. For example, recent 2025 meta-analyses report pooled hazard ratios for highest versus lowest TyG categories in the range of ~1.6–2.4 for MACEs and significant associations with myocardial infarction, stroke and CV death; a 2025 meta-analysis focused on METS-IR found higher METS-IR to be associated with increased composite CVD, CAD and stroke risk and described dose–response relationships with identifiable inflection points beyond which risk accelerates. Studies using eGDR likewise show lower eGDR (i.e., greater IR) predicting incident CVD in non-diabetic cohorts and improving model discrimination when added to conventional risk models. Importantly, several analyses adjusted for traditional risk factors (lipids, blood pressure, BMI, smoking) and still found independent associations, indicating that IR conveys residual cardiovascular risk not captured by standard risk calculators [40,43,44].

Several features of the recent literature merit emphasis. First, the relationship between IR and CVD often demonstrates nonlinearity or threshold effects: dose–response meta-analyses have identified METS-IR values above which incident risk rises disproportionately, suggesting clinically meaningful cut-points for risk stratification. Second, IR relates to cardiovascular outcomes even among persons without diagnosed diabetes, which argues for shifting the conceptual model from “diabetes-centric” risk to a broader “insulin-resistance continuum” model. Third, while multiple surrogates correlate with the euglycemic clamp (the research gold standard), simpler indices (TyG, METS-IR, TyG-BMI) perform well in large epidemiologic datasets and are pragmatic for clinical use; recent studies validate their prognostic value in general, hypertensive, renal and rheumatologic cohorts. Fourth, heterogeneity across studies remains (different populations, endpoints, cut-points), so pooled estimates should be interpreted with attention to clinical context and study design [3,45,46].

From a clinical and public-health standpoint, these mechanistic and epidemiologic data collectively support regarding insulin resistance as a clinically actionable, independent cardiovascular risk factor. Operationally this argument has three implications.

(1) Screening and risk stratification: incorporating validated IR surrogates (TyG, METS-IR, eGDR or HOMA-IR when insulin is measured) into routine cardiovascular risk assessment could reveal at-risk individuals who are normoglycaemic yet metabolically vulnerable, particularly when combined with imaging or inflammatory markers.
(2) Early multimodal intervention: identification of IR should prompt evidence-based lifestyle interventions (structured weight loss, exercise, dietary patterns that reduce hepatic de novo lipogenesis and improve insulin sensitivity) and consideration of pharmacologic agents with insulin-sensitizing or cardiometabolic benefits. Emerging data suggest that agents primarily studied for glucose lowering (SGLT2 inhibitors, GLP-1 receptor agonists) and older insulin-sensitizers (metformin, thiazolidinediones in selected contexts) can favorably modulate some pathways linked to IR (inflammation, oxidative stress, myocardial energetics), but randomized trials specifically targeting IR as the primary strategy to reduce cardiovascular events in non-diabetic populations are limited and represent a high-priority research need.
(3) Precision prevention and thresholds: the identification of nonlinear risk thresholds for METS-IR and similar indices suggests a potential for tiered prevention—people above certain index values may warrant more intensive intervention and follow-up [35,47].

Despite compelling progress, knowledge gaps remain and should guide future research. Standardization is needed regarding the optimal surrogate(s) of IR for clinical risk prediction (trade-offs between accuracy, availability and cost), and prospective randomized trials are required to determine whether interventions that specifically reduce IR (independent of glycaemic lowering) translate into fewer hard cardiovascular events in non-diabetic cohorts. Additionally, mechanistic human studies linking changes in IR with serial imaging of plaque biology or myocardial structure/function would clarify causal pathways and identify mechanistic biomarkers for early therapeutic response. Finally, population-level work must evaluate how best to implement IR screening in routine care, including cost-effectiveness and equity considerations, given the high global prevalence of metabolic dysfunction [48,49].

A growing body of mechanistic, imaging and epidemiologic evidence from 2023–2025 positions insulin resistance as a central mediator of cardiovascular pathology and an independent, actionable risk factor. Multiple validated surrogate indices (TyG, METS-IR, eGDR, HOMA-IR and derivatives) predict MACEs, MI, stroke and cardiovascular death across diverse populations, including non-diabetic individuals, and mechanistic studies elucidate how IR drives endothelial dysfunction, dyslipidemia, inflammation, oxidative stress, hypertension and myocardial metabolic derangement. Translating these insights into practice will require consensus on screening strategies, prospective trials targeting IR reduction for CVD prevention, and integration of IR assessment into multimodal cardiovascular risk management.

### 3.4. Insulin Resistance as a Biomarker Linking Metabolic Dysfunction with Structural and Functional Brain Alterations

#### 3.4.1. Insulin Resistance as a Systemic Metabolic Biomarker

Insulin resistance (IR) represents a core disturbance in the transition from metabolic health to overt disease, particularly in the context of obesity, prediabetes, and type 2 diabetes (T2D). Importantly, IR often develops early at the hepatic level, where impaired insulin-mediated suppression of gluconeogenesis disrupts fasting glycemic control and contributes to compensatory hyperinsulinaemia, even when postprandial glucose uptake in skeletal muscle and adipose tissue remains relatively preserved.

As insulin resistance progresses, impaired insulin signalling in adipose tissue and skeletal muscle leads to reduced peripheral glucose uptake and further aggravates hyperinsulinaemia. Early dysfunction in adipose insulin sensitivity is not an isolated event: it drives a cascade of metabolic disturbances, including dysregulated lipid handling, increased release of free fatty acids, chronic low-grade inflammation, and altered adipokine secretion from hypertrophic fat depots, particularly visceral adipose tissue.

In parallel, hepatic insulin resistance promotes increased hepatic lipid synthesis and very-low-density lipoprotein (VLDL) overproduction, linking impaired glucose regulation with dyslipidaemia. These combined alterations contribute to systemic metabolic stress, in which pro-inflammatory cytokines such as IL-6 and TNF-α and adipokines including elevated leptin and reduced adiponectin further exacerbate insulin signalling defects and establish a mechanistic link between metabolic and vascular pathology [50,51].

Clinically, IR is often quantified using biochemical indices that are accessible and informative in routine practice. The homeostasis model assessment for insulin resistance (HOMA-IR), calculated from fasting glucose and insulin levels, correlates strongly with physiological measures of insulin sensitivity and predicts early metabolic dysregulation in both non-diabetic and diabetic populations. Elevated HOMA-IR has been independently associated with subclinical vascular changes such as increased carotid intima-media thickness and prospective atherosclerotic risk, underscoring its value beyond glycaemic assessment alone [52].

Because fasting insulin is not always measured in primary care, lipid-based markers of IR have gained traction. The triglyceride-to-HDL cholesterol ratio (TG/HDL-C) shows consistent positive correlations with HOMA-IR across diverse populations, including overweight and obese children, and a higher TG/HDL-C ratio identifies individuals with more pronounced IR with good sensitivity. Moreover, large cohorts demonstrate that elevated TG/HDL-C, as well as related indices such as the triglyceride–glucose (TyG) index, are linked to cardiometabolic outcomes including heart failure and cardiovascular disease, suggesting that these simple ratios capture deleterious metabolic stress associated with insulin resistance [53,54].

Beyond TG/HDL-C, expanding research uses non-traditional lipid indices and multi-parameter scores (for example non-HDL cholesterol-to-HDL ratio, visceral adiposity measures or combined indices like TyG-BMI) to refine insulin resistance assessment and cardiometabolic risk stratification in large, population-based samples. These metrics integrate dyslipidemia, adiposity and glucose metabolism into composite biomarkers that can reflect the severity and progression of metabolic dysregulation more holistically than single measures alone [55].

Insulin resistance emerges as a systemic metabolic biomarker that captures the interplay between glucose homeostasis, lipid disturbances and chronic inflammation. Using a combination of HOMA-IR, fasting insulin, TG/HDL-C and other lipid indices allows clinicians and researchers to monitor the trajectory of metabolic dysregulation, identify individuals at high cardiometabolic risk early and tailor interventions accordingly.

#### 3.4.2. Effects of Insulin Resistance on Hippocampal Structure

Insulin resistance in the context of obesity and type 2 diabetes (T2D) is increasingly recognized as a contributor to structural changes in the hippocampus, a brain region essential for learning and memory. Multiple clinical neuroimaging studies have shown that individuals with obesity and T2D have smaller hippocampal volumes compared with matched non-diabetic controls, suggesting that chronic metabolic dysfunction is associated with loss of neuronal tissue in this area of the medial temporal lobe. In obese adolescents with T2D, for example, hippocampal volume reductions have been documented relative to obese but non-diabetic peers, indicating that altered glucose and insulin handling early in life may influence neural integrity [56].

At the cellular and molecular level, impaired insulin and insulin-like growth factor-1 (IGF-1) signaling—hallmarks of systemic insulin resistance—disrupt intracellular pathways that support synaptic plasticity and neuron survival. Insulin and IGF-1 receptors are highly expressed in hippocampal neurons, and their downstream PI3K/Akt and MAPK cascades normally facilitate long-term potentiation and structural maintenance of synapses. When signaling through these receptors is diminished, as occurs in obesity and chronic hyperglycemia, markers of synaptic plasticity decline, dendritic arbor complexity decreases, and processes such as adult neurogenesis are reduced, all of which contribute to atrophy and impaired learning [9,57,58].

Experimental models further clarify mechanisms by which insulin resistance alters hippocampal structure. In rodents, diet-induced peripheral and central insulin resistance impairs hippocampal long-term potentiation and reduces dendritic spine density, consistent with fewer functional synapses, and these changes coincide with deficits in spatial memory tasks. Hippocampal-specific induction of insulin resistance likewise leads to reduced numbers of immature neurons and diminished dendritic complexity, reinforcing the idea that insulin signaling directly supports hippocampal structural integrity independent of systemic metabolic state [59,60].

A related consequence of impaired insulin action is altered glucose uptake in neurons. In T2D and insulin-resistant subjects, both reduced cerebral glucose metabolism and diminished hippocampal responsiveness to insulin have been observed, linking metabolic inflexibility to cognitive dysfunction. Chronic hyperglycemia and hyperinsulinemia can further exacerbate this problem by promoting oxidative stress and inflammatory changes that blunt glucose transporter expression in the brain, weakening energy supply to the hippocampus and contributing to synaptic failure and memory impairment [61].

Together, these lines of evidence indicate that insulin resistance—through disrupted insulin/IGF-1 signaling, altered cerebral glucose handling, and downstream effects on synaptic plasticity and neurogenesis—is not merely a peripheral metabolic disturbance but a factor capable of reshaping hippocampal structure and compromising the neural substrates of memory, particularly in individuals with long-standing obesity and type 2 diabetes.

#### 3.4.3. Impaired Brain Glucose Metabolism and Disrupted Insulin Signaling

Impaired brain glucose metabolism is a robust finding in people with obesity and type 2 diabetes (T2D), and this deficit appears to track with systemic insulin resistance. Positron emission tomography using [^18^F]fluorodeoxyglucose ([^18^F]FDG-PET) indicates that cerebral glucose uptake in individuals with higher HOMA-IR may differ from that observed in insulin-sensitive controls, with patterns that vary according to brain region, disease stage, and compensatory metabolic mechanisms, even in the absence of overt cognitive impairment.

These hypometabolic signatures are not uniform across the brain: medial temporal lobe structures, including the hippocampus, and frontal regions involved in memory and executive function are particularly affected, linking metabolic dysfunction to regional vulnerability. In elderly cohorts, higher peripheral insulin resistance predicts lower FDG uptake in these key networks, suggesting that systemic metabolic stress translates into inadequate neuronal energy supply.

At the molecular level, the convergence of chronic hyperglycemia and peripheral insulin resistance in T2D disrupts canonical insulin and insulin-like growth factor-1 (IGF-1) signaling pathways in neurons and glia. Insulin and IGF-1 receptors, highly expressed in the hippocampus, trigger downstream cascades such as PI3K-Akt and MAPK that are critical for neuronal survival, synaptic plasticity and energy homeostasis [62]. In the insulin-resistant state, impaired activation of PI3K-Akt not only weakens glucose transporter trafficking and neuronal glucose utilization, but also removes inhibitory control over glycogen synthase kinase-3β (GSK-3β). Unchecked GSK-3β activity has been linked to tau hyperphosphorylation and microtubule destabilization in human and animal models, connecting metabolic signaling defects to pathological hallmarks of neurodegenerative processes. Moreover, chronic impairment of these pathways reduces trophic support and increases susceptibility to oxidative stress and mitochondrial dysfunction, creating a sustained energy deficit at the cellular level [63,64].

**Figure 2:**
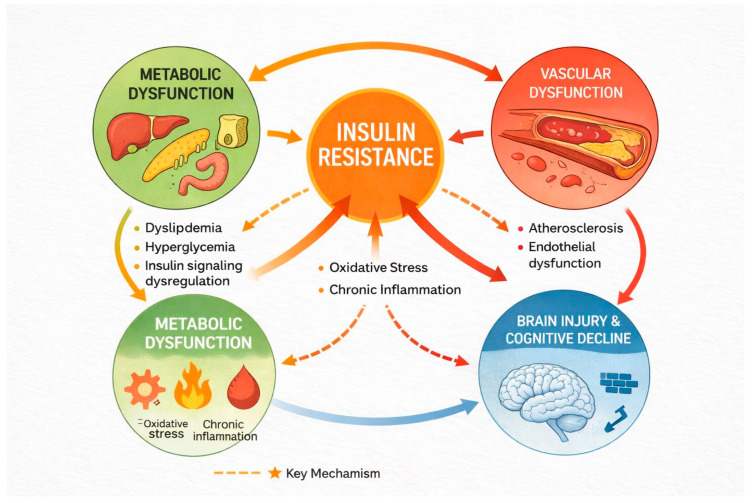
How Insulin Resistance Impacts Brain Function. The graphic depicts the cardio–metabolic–brain axis as an interconnected network. It shows insulin resistance at the center, linking metabolic problems—like impaired glucose handling and lipid imbalance—to vascular issues such as endothelial dysfunction.

The metabolic milieu typical of obesity and T2D—marked by hyperglycemia, insulin resistance and dyslipidemia—further exacerbates neuropathological risk by interacting with amyloid and tau protein dynamics. Dyslipidemia promotes peripheral and central inflammation, alters lipid raft composition in neuronal membranes and impairs clearance mechanisms for amyloid-β peptides, facilitating their aggregation. In parallel, hyperglycemia induces advanced glycation end-products (AGEs) and oxidative damage that accelerate tau pathology and destabilize synapses. Collectively, these processes create a brain environment in which energy deficits, disrupted signaling and proteinopathy feed into each other, increasing the likelihood of cognitive decline and neurodegenerative change. The idea that metabolic dysfunction may serve as a driving force in at least some forms of dementia has been encapsulated in concepts such as “type 3 diabetes,” reflecting insulin resistance within the central nervous system as a contributor to Alzheimer’s-like pathology [65]. As illustrated in Figure 2, the cardio–metabolic–brain axis represents a network of interconnected pathways through which insulin resistance links metabolic dysfunction with vascular impairment and brain injury. Reduced insulin sensitivity promotes chronic inflammation, oxidative stress, and endothelial dysfunction, ultimately leading to impaired cerebral perfusion and disrupted neuronal metabolism, thereby increasing vulnerability to cognitive decline.

#### 3.4.4. Altered Functional Brain Network Organization

In individuals with obesity or type 2 diabetes (T2D), systemic insulin resistance is associated with widespread alterations in the functional organization of large-scale brain networks, reflecting reduced efficiency of neural communication and disrupted integration among regions supporting executive and memory functions. Resting-state functional magnetic resonance imaging (rs-fMRI) studies consistently show that higher levels of insulin resistance correlate with weaker functional connectivity within the default mode network (DMN), as well as between DMN hubs and regions implicated in executive control, such as prefrontal and temporal cortices. In T2D patients, this dysconnectivity within the DMN—including reduced coherence between the posterior cingulate cortex and middle temporal gyrus—has been linked to both elevated HOMA-IR and lower performance on memory and executive tests, suggesting that network disruption may be a neurobiological substrate for emerging cognitive difficulties [66,67].

Beyond DMN abnormalities, obesity itself is associated with altered resting-state connectivity patterns. Obese adults show changes in functional coupling strength not only in DMN regions such as the precuneus and medial prefrontal cortex, but also in temporal and reward-related circuits, and these alterations are correlated with indices of insulin resistance and fasting insulin levels, indicating that metabolic dysfunction can modulate intrinsic brain network architecture even in the absence of overt diabetes [68].

Graph theory and connectome analyses provide a more integrative view of these network changes. In both T2D patients with mild cognitive impairment and in older adults with obesity, measures of global and local network topology derived from rs-fMRI reveal disruptions in efficiency and clustering that reflect a less optimized balance between functional segregation and integration. Some studies report reduced global efficiency alongside shifts in nodal efficiency within default mode and executive network regions, a pattern that may reflect compensatory reorganization in early disease phases but ultimately portends cognitive decline as network topology becomes increasingly inefficient [69].

Furthermore, newer work using advanced network topology methods shows that higher peripheral insulin resistance is associated with altered network metrics in hippocampal and executive circuits, linking insulin resistance not just to isolated connectivity changes but to broader topological reorganization of the functional connectome. Such reconfiguration—including diminished capacity for information integration across distant brain regions—is predictive of future cognitive decline and may mediate the vulnerability of memory and executive systems in chronic obesity and T2D [70].

These findings support the view that metabolic stress from insulin resistance has measurable impacts on the brain’s functional network organization, disrupting the coordinated dynamics of key cognitive systems and potentially setting the stage for progressive cognitive impairment.

#### 3.4.5. Neuroinflammation and Oxidative Stress as Mediators

In obesity and type 2 diabetes, chronic low-grade systemic inflammation extends into the central nervous system, where it acts as a key mediator of neural dysfunction. Excess adiposity and metabolic overload promote activation of resident immune cells in the brain, especially microglia and astrocytes, leading to sustained production of pro-inflammatory cytokines such as TNF-α, IL-1β and IL-6 and the disruption of the blood–brain barrier, facilitating further immune cell infiltration and neuroinflammatory signalling. These processes are evident in rodent models of diet-induced obesity and in clinical cohorts with metabolic disease, where microglial activation closely tracks both measures of systemic insulin resistance and indices of neuroinflammation [71].

Insulin resistance itself contributes to oxidative stress and mitochondrial dysfunction, creating a self-reinforcing cycle of cellular damage in neural tissue. In the insulin-resistant brain, elevated levels of glucose and free fatty acids fuel excess generation of reactive oxygen species (ROS), which overwhelm endogenous antioxidant defenses and impair mitochondrial oxidative phosphorylation. This redox imbalance not only damages cellular proteins and membranes but also activates pro-inflammatory transcription factors such as NF-κB, further amplifying inflammatory responses and compromising neuronal survival and function. These oxidative and inflammatory processes provide a direct link between metabolic dysregulation and cognitive decline, showing how insulin resistance can compromise brain health. These mechanisms are supported by evidence showing that obesity-related oxidative stress is linked to impaired glucose transporter function, increased ROS production and exacerbated neuroinflammation in both preclinical models and human studies. Targeting oxidative stress pathways can reduce neuroinflammation and help preserve neuronal function, emphasizing the potential for therapeutic strategies to prevent cognitive impairment in metabolic disorders [35,72].

Importantly, chronic metabolic inflammation in obesity and T2D serves as a critical bridge between peripheral metabolic dysfunction, vascular injury and neurodegenerative processes. Neuroinflammation and oxidative stress interact to disrupt cellular energy homeostasis, promote mitochondrial impairment and facilitate accumulation of pathological protein species, contributing to synaptic loss and cognitive decline. The convergence of these processes is increasingly implicated in the pathogenesis of diabetic encephalopathy and Alzheimer’s disease, with both preclinical and clinical data indicating that elevated inflammatory mediators and ROS precede measurable neurodegeneration in vulnerable brain regions [73].

#### 3.4.6. Cardiometabolic Risk and Neural Vulnerability

Insulin resistance (IR) in the context of obesity and type 2 diabetes (T2D) extends its detrimental effects beyond metabolic tissues to the vasculature, where it impairs endothelial function and undermines cerebral perfusion, creating a vulnerable environment for cognitive structures. Under normal conditions, insulin stimulates endothelial nitric oxide production via the PI3K/Akt pathway, promoting vasodilation and maintaining microvascular perfusion. In insulin-resistant states these pathways are disrupted, leading to endothelial dysfunction, reduced nitric oxide availability, increased arterial stiffness and a pro-atherogenic milieu that compromises dynamic regulation of cerebral blood flow (CBF) and nutrient delivery to the brain [74].

Neuroimaging studies support these mechanistic links by showing that individuals with IR or T2D exhibit reduced cerebral perfusion, particularly in regions such as the posterior cingulate cortex and precuneus that are critical for memory and cognitive processing. Using arterial spin-labeling MRI, hypoperfusion in these areas correlates with higher insulin resistance and is associated with poorer cognitive performance, indicating that impaired vascular supply may precede or contribute to early cognitive dysfunction in metabolic disease [75].

Beyond global hypoperfusion, IR and metabolic dysregulation also disrupt cerebral autoregulation and microvascular integrity, leading to chronic microischemia and impaired neurovascular coupling. Microvascular changes in T2D and obesity—driven by hyperglycemia, hypertension and IR—include increased blood–brain barrier permeability, endothelial and pericyte dysfunction, and hypoxia at the capillary level. These vascular alterations compromise the ability of cerebral vessels to adjust blood flow to local metabolic demands, increasing the susceptibility of neural tissue to ischemic injury, white matter lesions and small infarcts [76].

Importantly, the burden of metabolic and vascular dysfunction in obesity and T2D aligns with the concept of a cardio–metabolic–brain axis, wherein IR serves as a shared pathophysiological denominator linking peripheral metabolic disorders with cardiovascular disease and brain aging. Insulin resistance accelerates atherosclerosis, promotes arterial stiffness and interacts with other cardiometabolic risk factors such as dyslipidemia and hypertension, thereby amplifying cerebrovascular strain and promoting structural and functional brain decline over time. This integrated perspective helps explain why metabolic risk increases the likelihood of stroke, cognitive impairment and dementia, highlighting the interconnected nature of cardiometabolic and neural vulnerability [77].

#### 3.4.7. Clinical Implications

Insulin resistance is increasingly recognized as an independent risk factor for cognitive decline, mild cognitive impairment (MCI) and dementia, even in populations without overt type 2 diabetes (T2D). Longitudinal cohort analyses and metabolic profiling studies have shown that higher levels of IR, typically assessed by HOMA-IR, are associated with both poorer performance in memory and executive domains and with progression from MCI to Alzheimer’s disease dementia, underscoring that metabolic dysregulation contributes to neural vulnerability beyond glucose intolerance alone. In a well-characterized prospective cohort, a plasma metabolic signature reflecting insulin resistance predicted conversion from MCI to Alzheimer’s dementia, suggesting that IR can serve as an early harbinger of neurodegenerative progression [78].

Beyond cognitive outcomes, higher HOMA-IR has been linked with worse scores on measures of verbal memory, executive function and global cognition in cognitively normal adults, and with elevations in cerebrospinal tau biomarkers that are indicative of neurodegenerative processes, supporting the idea that IR contributes to subclinical neural injury well before clinical dementia manifests [79].

From a clinical perspective, IR-related indices such as HOMA-IR, lipid parameters (e.g., triglyceride-to-HDL ratios) and circulating markers of inflammation and adipose tissue dysfunction offer pragmatic tools for early risk stratification. These biomarkers reflect underlying metabolic and inflammatory pathways that are mechanistically linked to brain aging and neurodegeneration, making them useful in identifying individuals at elevated risk of cognitive decline in the context of metabolic disorders [80].

Importantly, interventions that improve insulin sensitivity and address cardiometabolic risk have shown promise not only for metabolic and cardiovascular outcomes but also for neuroprotection. Glucagon-like peptide-1 receptor agonists (GLP-1RAs) have demonstrated cognitive benefits in patients with T2D, preserving functional connectivity and slowing decline in some trials, and are under investigation for their effects on MCI and dementia in broader populations [81,82]. Metformin, a first-line antihyperglycemic agent, has been linked with better memory and executive function in observational studies and has shown beneficial effects on microglial homeostasis, mitochondrial turnover and neuroinflammation in preclinical models, pointing to mechanisms beyond glycemic control that may protect neuronal networks. Additional classes of drugs used in T2D—including SGLT2 inhibitors and DPP-4 inhibitors—exhibit systemic anti-inflammatory and metabolic effects that could contribute to reduced cognitive risk, although evidence from dedicated neurocognitive trials is still emerging [83]. Furthermore, non-pharmacological approaches such as structured weight loss and regular physical activity improve insulin sensitivity and are associated with better cognitive outcomes in observational and interventional research, emphasizing that metabolic health optimization may confer neuroprotective effects across the lifespan.

### 3.5. Insulin Resistance and Cognitive Function

Insulin reaches the brain through a regulated, receptor-mediated transport mechanism across the blood–brain barrier, which ensures controlled entry into the central nervous system [84]. Once inside, insulin binds to neuronal and glial insulin receptors, activating intracellular signaling cascades that support synaptic plasticity, neuronal metabolism, and neurotransmission [85]. When insulin signaling becomes impaired, the brain’s ability to utilize glucose efficiently declines, leading to reduced energy availability in regions critical for learning and memory [86]. A contributing mechanism is the downregulation of glucose transporter proteins, including GLUT4, in insulin-responsive neurons. Reduced expression of GLUT4 has been reported in hippocampal neurons of subjects with type 2 diabetes, supporting the notion that defects in glucose uptake occur not only through signaling pathways but also at the level of transport into cells. These metabolic disturbances manifest as measurable deficits in cognitive performance, particularly in memory formation, executive functioning, and learning processes [87]. In parallel, central insulin resistance promotes neuroinflammatory activation and contributes to pathological amyloid-β accumulation, processes strongly associated with neurodegenerative pathways resembling those observed in Alzheimer’s disease. Neuroimaging studies further demonstrate that higher levels of insulin resistance correlate with reduced hippocampal volume and disrupted functional network connectivity, linking metabolic dysfunction to observable structural and functional brain changes [88].

Reduced glucose utilization in the brain also becomes evident in altered relationships between glycemic measures and cognitive performance, including associations between fasting glucose and specific cognitive domains, emphasizing that both systemic and neuronal glucose handling are important for cognitive health [87,89,90].

Brain insulin resistance disrupts the signaling pathways that normally support neuronal metabolism, which promotes oxidative stress, inflammation, and impaired insulin receptor activity—processes that together create conditions conducive to early Alzheimer-related changes [91]. As insulin signaling becomes dysfunctional, neuronal glucose utilization declines, and this metabolic stress accelerates amyloid accumulation, tau alterations, and synaptic vulnerability, linking diabetes-related dysregulation with mechanisms that drive cognitive decline [92]. Insulin resistance further amplifies Alzheimer pathology by enhancing amyloid-β production, reducing its clearance, and promoting tau hyperphosphorylation, establishing a biochemical bridge between the two hallmark lesions of the disease [93]. Ultimately, chronic impairment of insulin action in the brain triggers a cascade involving mitochondrial dysfunction, lipid dysregulation, and neuroinflammation, explaining how insulin resistance can initiate or intensify Alzheimer-type neurodegeneration even at its earliest stages [94].

Higher HOMA-IR values in young adults are associated with subtle reductions in memory performance and with structural alterations in medial temporal and prefrontal regions, indicating that insulin resistance can influence cognition and brain morphology even at an early age [95]. Among young men, increased adiposity is linked to elevated insulin and HOMA-IR levels, and these metabolic disturbances correspond to poorer executive functioning and diminished working-memory performance, suggesting that insulin resistance mediates part of the cognitive impact of excess body fat [96]. In older adults without neurological disease, higher HOMA-IR is strongly associated with slower processing speed and deficits in specific executive functions, showing a selective vulnerability of frontal cognitive networks to impaired insulin signaling [97]. Middle-aged individuals with higher HOMA-IR—often presenting with non-alcoholic fatty liver disease and greater liver fibrosis—also demonstrate reduced performance in domains such as attention and executive function, reinforcing the link between systemic insulin resistance and cognitive decline [57]. Clinical studies in patients with diabetes consistently show that elevated HOMA-IR correlates with lower scores in verbal learning, attention, and global cognition, supporting the role of insulin resistance as a measurable biomarker associated with progressive cognitive impairment [98].

Viewing Alzheimer’s disease through the framework of “type 3 diabetes” emphasizes that elevated HOMA-IR reflects systemic metabolic disturbances that compromise neuronal energy homeostasis, increase oxidative and inflammatory stress, and heighten vulnerability to early cognitive decline and dementia [99]. Long-term metabolic observations further indicate that impaired glucose regulation—including higher HOMA-IR—is associated with greater tau burden on PET imaging years later, suggesting that insulin resistance may accelerate neurodegenerative processes linked to cognitive deterioration [100]. Disruptions in insulin signalling also weaken hippocampal functions essential for learning and memory, implying that individuals with elevated HOMA-IR may face a heightened risk of developing mild cognitive impairment and Alzheimer-related cognitive deficits [101]. Pathological tau species additionally interfere with components of the neuronal insulin-signalling cascade, leading to impaired glucose utilization and further cognitive decline, thereby reinforcing the connection between insulin resistance and progression toward dementia [39].

Insulin signalling in the hippocampus plays a critical role in supporting neuronal metabolism, synaptic plasticity, and memory formation, and disruptions in this pathway link insulin resistance to hippocampal dysfunction characteristic of Alzheimer’s disease [101]. Peripheral and central insulin resistance further contribute to cognitive impairment by altering brain glucose utilization, weakening insulin receptor activity, and disturbing network-level processes that support learning and executive function [35]. Middle-aged adults with impaired glucose metabolism also demonstrate smaller hippocampal volumes, indicating that metabolic dysregulation manifests not only as functional vulnerability but also as structural brain changes [102]. In older adults, elevated insulin resistance is associated with altered functional brain network topology, including reduced integration and efficiency of cognitive networks, which may mediate the link between metabolic dysfunction and cognitive decline [68]. Additional imaging analyses reveal that higher insulin resistance corresponds to disrupted connectivity in key resting-state networks, suggesting that metabolic impairment influences large-scale brain organization long before clinical symptoms appear [103].

### 3.6. Heart Disease as a Mid-Pathway Factor

Peripheral insulin resistance (IR) represents a fundamental metabolic disturbance that—even before the formal diagnosis of diabetes—predisposes individuals to the development of cardiovascular diseases (CVDs), including hypertension, atherosclerosis and heart failure. The literature repeatedly shows that IR promotes dyslipidemia (elevated triglycerides, increased LDL-C, the presence of small dense LDL particles) and disrupts the regulation of glucose and lipid metabolism, which leads to chronic hyperglycemia, increased release of free fatty acids from adipose tissue and, consequently, persistent oxidative stress and inflammation [104,105,106].

At the same time, IR impairs the function of the vascular endothelium and vascular smooth muscle cells (VSMCs), resulting in reduced nitric oxide (NO) production, impaired vascular relaxation, increased arterial stiffness, diminished adaptability of blood flow and a lowered vascular reserve [107]. As a result, this promotes the development of macro- and microangiopathy, the formation of atherosclerotic plaques and vascular narrowing, which limits the ability of the circulatory system to efficiently deliver oxygen and nutrients to tissues—including those within the brain [108,109].

In the context of the brain, these vascular abnormalities—chronic inflammation, oxidative stress, large-vessel atherosclerosis and microangiopathy—can substantially reduce blood flow in regions critical for cognitive function, such as the hippocampus, prefrontal cortex and subcortical structures [110,111]. This leads to weakened cerebral autoregulation and reduced vascular reserve, making the brain more vulnerable to hemodynamic, metabolic or oxidative fluctuations. Research indicates that insulin resistance plays a central role in these vascular changes by promoting endothelial dysfunction and accelerating atherosclerotic processes, which in turn impair cerebral perfusion. In addition, population studies have shown a clear link between insulin resistance and cognitive decline in older adults, supporting the connection between metabolic dysfunction and reduced brain function.

Empirical data support this chain of associations: in the InCHIANTI cohort study, individuals without diabetes but showing signs of insulin resistance had a higher risk of cognitive impairment with features of subcortical vascular injury, suggesting that IR may mediate cognitive decline through vascular mechanisms. Review analyses also indicate that IR or metabolic syndrome increases the risk of neurodegenerative diseases, including Alzheimer’s disease (AD), not only through direct intra-brain disturbances (such as brain insulin resistance, impaired glucose metabolism, accumulation of β-amyloid or tau) but also through chronic neuroinflammation, oxidative stress, and compromised perfusion and vascular reserve [108].

In this light, one can propose a “domino effect”: IR → development of CVD (hypertension, atherosclerosis, heart failure and/or small-vessel disease) → deterioration of cerebral circulation (reduced perfusion, diminished vascular reserve, impaired autoregulation) → gradual decline in cognitive function, including deficits in memory, attention and processing speed—which over time may contribute to the development of mild cognitive impairment (MCI) or dementia [112].

Furthermore, an increasing number of mediation studies and reviews show that vascular and cardiac diseases are not merely coexisting comorbidities in individuals with IR, but in fact represent an important intermediate link in the pathological sequence connecting metabolic dysfunction to cognitive impairment or neurodegeneration [113,114].

For this reason—both from a research and clinical perspective—IR should be viewed as a modifiable cerebrovascular and neurocognitive risk factor. Interventions aimed at reducing insulin resistance, improving the lipid profile, lowering inflammation, preventing atherosclerosis or enhancing endothelial function may not only have cardiovascular or metabolic benefits, but may also slow or delay cognitive decline.

### 3.7. Foundational Mechanisms from Metabolic Imbalance to Neurodegeneration

Chronic metabolic dysregulation—particularly persistent hyperglycemia accompanied by hyperinsulinemia and insulin resistance—constitutes the initiating point for long-term, multi-level disturbances affecting both the vascular and nervous systems. Excess glucose in the bloodstream and within cells overloads metabolic pathways: excessive glycolysis and accumulation of intermediate metabolites promote the formation of reactive oxygen species (ROS), while mitochondria become less efficient, leading to impaired energy production and increased oxidative stress. This, in turn, favors the formation of advanced glycation end-products (AGEs), which can damage proteins, lipids, and receptors on endothelial cells, microglia, and astrocytes, activating pro-inflammatory pathways via receptors such as RAGE. In conditions of insulin resistance, even in the presence of excess circulating insulin, intracellular insulin signaling (e.g., through IRS-1 → PI3K → Akt) becomes impaired, disrupting neuronal glucose metabolism and energy homeostasis, and affecting critical neuronal survival functions such as protein synthesis, kinase/phosphatase regulation, autophagy, and antioxidant defense mechanisms. These disturbances increase the vulnerability of neurons and glial cells to subsequent damage [115].

At the vascular level, insulin resistance and metabolic disturbances exert deleterious effects on endothelial cells, both peripheral and cerebral. Cerebral microvascular endothelial cells, together with pericytes and astrocytes, form the core components of the blood–brain barrier (BBB), regulating the entry of substances from the blood into the brain. Endothelial insulin receptors not only facilitate insulin transcytosis into the brain but also participate in signaling pathways that regulate barrier function, vascular tone, and vascular homeostasis [116,117]. In the context of insulin resistance, growing evidence indicates that dysfunction of the blood–brain barrier (BBB) plays a key role in limiting insulin access to the central nervous system. Alterations in endothelial structure and transport mechanisms within cerebral microvessels, including impaired caveolae-dependent transcytosis, are associated with reduced insulin transport across the BBB. As a consequence, insulin delivery to the brain parenchyma is diminished, leading to impaired central insulin signaling. BBB dysfunction in metabolic and neurodegenerative conditions is further characterized by increased permeability, endothelial inflammation, and oxidative stress, which together contribute to an unfavorable cerebral microenvironment. These alterations can exacerbate neuronal vulnerability by disrupting metabolic support and promoting inflammatory signaling within the central nervous system, thereby linking peripheral insulin resistance with impaired brain homeostasis and increased susceptibility to neurodegenerative processes [118,119].

In addition to impaired signaling, endothelial dysfunction results in reduced nitric oxide (NO) production via eNOS, decreasing vasodilatory capacity, impairing hemodynamics, and disrupting cerebral microcirculation. While many studies focus on peripheral vascular changes in diabetes and insulin resistance, analogous mechanisms in cerebral microvessels may compromise perfusion, neurovascular coupling, and energy delivery in metabolically demanding regions such as the hippocampus, prefrontal cortex, and white matter. In animal models, insulin resistance leads to decreased eNOS activity, diminished NO synthesis, and impaired vascular responses to metabolic stimuli [69].

On this background, chronic inflammation and oxidative stress emerge. Excess glucose, free fatty acids, and other metabolites, combined with insulin dysfunction, stimulate the production of pro-inflammatory cytokines (e.g., TNF-α, IL-1β, IL-6) and activate signaling pathways such as NF-κB, JNK, and PKC in endothelial cells, microglia, and astrocytes [120]. Microglia and astrocytes adopt a reactive phenotype, releasing ROS, NO, and cytokines, and amplifying glycation processes through RAGE activation, all of which exacerbate the neurotoxic environment [121]. In these conditions, synaptic homeostasis is disrupted: metabolic support for neurons declines, glutamate clearance is impaired, susceptibility to oxidative stress increases, and organelle damage—including mitochondrial dysfunction—may culminate in neuronal apoptosis.

Simultaneously, these processes compromise blood–brain barrier integrity. Both animal models of high-fat/high-sugar diet-induced insulin resistance and clinical studies demonstrate that endothelial dysfunction, oxidative stress, and inflammation reduce the expression of tight junction proteins (e.g., claudins, occludin, ZO-1), resulting in increased BBB permeability [101,122,123]. In pre-diabetic mouse models, early BBB destabilization and activation of astrocytes and microglia occur even before cognitive deficits become apparent. With continued dietary and metabolic stress, neurodegeneration develops alongside worsening cognitive performance, correlating with progressive barrier dysfunction.

Once the barrier is compromised, pro-inflammatory cytokines and excess metabolites more readily enter the brain, and glucose and insulin transport becomes less controlled, aggravating brain insulin resistance (BIR). Reduced insulin availability in the CNS and impaired intracellular insulin signaling weaken neuroprotective mechanisms, including protein production and degradation, synaptic homeostasis, autophagy, and ROS detoxification.

Within this energetically, oxidatively, inflammatory, and vascularly compromised environment, neurons and other brain cells are especially vulnerable to pathology. Impaired insulin signaling and reduced glucose availability produce energy deficits that hinder maintenance of complex neuronal functions, including synaptic integrity, axonal transport, protein synthesis, and damage repair. Concurrent chronic oxidative stress and kinase activation (e.g., GSK-3β, JNK) promote tau hyperphosphorylation and amyloid-β accumulation, while impaired clearance mechanisms exacerbate their deposition [124]. Aggregated Aβ and pathological tau disrupt synaptic function, intracellular transport, plasticity, and ultimately neuronal survival, while activated glia intensify neuroinflammation, perpetuating the degenerative cycle.

In light of these observations, the cardio–metabolic–brain axis emerges as a dynamic, multi-layered process in which metabolic disturbances (hyperglycemia, hyperinsulinemia/insulin resistance), endothelial dysfunction, inflammation and oxidative stress, BBB breakdown, and neurodegeneration are tightly interconnected and mutually reinforcing. Each element amplifies the next, forming a pathological loop that leads to impairment of both cognitive function and cardiovascular regulation. Clinically, this explains why individuals with metabolic syndrome, type 2 diabetes, or obesity frequently exhibit cognitive decline alongside increased cardiovascular risk.

These data further suggest that therapeutic or preventive interventions that improve insulin sensitivity, support endothelial function, reduce oxidative stress and inflammation, and maintain BBB integrity may confer dual benefits—both neuroprotective and cardioprotective [125]. Implementation of such strategies—via pharmacotherapy, diet, physical activity, modulation of inflammation, or barrier-protective factors—could be crucial in preventing or slowing cognitive decline and cardiometabolic disease progression.

### 3.8. Clinical Evidence: Metabolic and Neurocognitive Consequences of Insulin Resistance

Clinical data consistently show that insulin resistance acts as a key biomarker linking metabolic disturbances with measurable changes in brain structure and function. Evidence from young adults demonstrates that higher fasting glucose and HOMA-IR are associated with poorer cognitive performance and reduced brain volumes, indicating that metabolic dysregulation can influence neurocognitive processes even early in life [95]. These findings align with observations in diabetic populations, where elevated insulin levels, dyslipidemia, and pro-inflammatory biomarkers are closely related to the progression of cognitive decline, highlighting their relevance as clinical indicators of neurocognitive risk [98]. Structural imaging further supports this association, as older adults with impaired glucose metabolism exhibit smaller hippocampal volumes despite preserved cognition, suggesting that insulin resistance contributes to subclinical neurodegenerative changes [102]. Functional MRI studies extend this pattern by showing that type 2 diabetes is characterized by disrupted functional connectivity and alterations in network topology, linking metabolic abnormalities to impaired coordination of large-scale brain systems [121]. In addition, higher HOMA-IR clearly increases the likelihood of mild cognitive impairment among elderly individuals with T2DM, reinforcing insulin resistance as an important predictor of early cognitive deterioration [126]. Even in cognitively normal adults, elevated IR is associated with deficits in specific cognitive domains and with increased CSF tau concentrations, suggesting a mechanistic bridge between metabolic dysfunction and the earliest stages of neurodegeneration [127]. Complementary evidence from population studies indicates that higher triglyceride–glucose (TyG) index values are associated with an increased risk of cognitive impairment, underscoring the potential utility of TyG as an accessible metabolic biomarker with neurocognitive relevance [128]. Moreover, studies in aging adults suggest that insulin homeostasis mediates the association between metabolic fitness and processing speed, positioning insulin resistance as an intermediary process through which systemic metabolic health may influence cognitive performance [129]. Finally, longitudinal data indicate that insulin resistance is associated with subsequent cognitive decline in non-demented adults even when Alzheimer’s disease cerebrospinal fluid biomarkers remain stable, suggesting that metabolic pathways may contribute to neurocognitive deterioration independently of classical AD pathology [130].

### 3.9. Biomarkers Linking Metabolic Dysfunction, Cardiovascular Pathology and Brain Health

Evidence linking insulin resistance to neurodegeneration highlights that impaired insulin signaling is associated with reduced neuronal metabolic function, increased amyloidogenic processes, and heightened vulnerability of brain regions such as the hippocampus, positioning IR as a key metabolic biomarker influencing brain health [121]. Complementing these findings, FDG-PET studies in individuals with type 2 diabetes show distinct hypometabolic patterns in cortical and subcortical regions, providing an imaging-based neural biomarker of altered brain glucose utilization in the context of systemic metabolic dysfunction [131]. Blood-based biomarkers are similarly influenced by metabolic status, as factors such as hyperinsulinemia, inflammation, and dysregulated lipid metabolism modulate circulating levels of tau, amyloid-β, and neurofilament light chain, linking metabolic dysregulation with biochemical indicators of neurodegeneration [99]. A comprehensive review of in vivo biomarkers of brain insulin resistance further demonstrates that both laboratory markers (insulin, HOMA-IR, inflammatory cytokines) and neuroimaging methods (FDG-PET, fMRI, CSF analysis) collectively capture the impact of impaired insulin signaling on neural systems, underscoring the need for multimodal biomarker assessment [132]. This metabolic–neural interface is reinforced by evidence showing that overnutrition and obesity promote IR-related neuroinflammation and oxidative stress, with adipokines and cytokines acting as measurable indicators of the metabolic and immune disturbances contributing to cognitive vulnerability [1]. Reviews of type 2 diabetes pathology additionally highlight that cardiometabolic abnormalities—including endothelial dysfunction, impaired glucose metabolism, and heightened oxidative and inflammatory stress—drive structural and functional changes in the brain, linking vascular biomarkers such as hs-CRP and adhesion molecules with neural outcomes [133]. Circulating biomarkers associated with early Alzheimer’s disease further demonstrate that metabolic and inflammatory mediators, including insulin, adipokines, cytokines, and lipid markers, contribute to changes in Aβ, tau, and NfL levels, illustrating how metabolic dysfunction is reflected in blood-based neural biomarkers [134]. Functional neuroimaging studies extend this framework by showing that higher insulin resistance correlates with disrupted network organization and altered connectivity in cognitive networks, indicating that biomarkers derived from fMRI can sensitively detect the neural consequences of metabolic impairment [135]. Microglia-focused biomarker research reveals that inflammation-related molecules such as IL-6, TNF-α, and CRP, in combination with insulin resistance and vascular dysfunction, form an interconnected set of measurable predictors that reflect the combined metabolic and neuroinflammatory burden driving cognitive decline [136]. Finally, clinical and molecular evidence confirms that metabolic and neural biomarkers converge in insulin-resistant states, as impaired insulin signaling measured in peripheral tissues parallels disruptions in neuronal pathways associated with amyloid processing, tau phosphorylation, and synaptic integrity, illustrating the integrative role of IR in cardiometabolic and neurodegenerative processes [68].

### 3.10. Therapeutic and Preventive Strategies Targeting Insulin Resistance Across Metabolic and Brain Health

Lifestyle interventions, particularly regular physical activity, have been shown to improve peripheral insulin sensitivity while simultaneously exerting beneficial effects on brain metabolism and cognitive performance, likely through enhanced glucose utilization, mitochondrial function, and neurotrophic signaling [137]. Experimental and clinical evidence further indicates that exercise can counteract insulin-resistance-related mitochondrial dysfunction within the brain, suggesting a mechanistic link between improved metabolic flexibility and preserved neuronal energy homeostasis [138]. Dietary modification combined with physical activity also favorably influences laboratory metabolic parameters, including fasting insulin and lipid profiles, thereby reducing systemic metabolic stress that may contribute to downstream vascular and neural impairment [139].

Pharmacological approaches targeting insulin resistance provide additional therapeutic potential. Metformin therapy in individuals with type 2 diabetes has been associated with improved metabolic control and, in some cohorts, better cognitive performance, supporting its relevance beyond glycemic regulation [140]. Mechanistic studies indicate that metformin may exert direct effects in the central nervous system by modulating neuroinflammation, oxidative stress, and insulin signaling pathways, which are implicated in neurodegenerative processes [141].

Bariatric surgery represents a particularly effective intervention for severe obesity and insulin resistance, leading to profound and sustained improvements in insulin sensitivity, adipokine secretion, inflammatory cytokine levels, and lipid profiles [142]. Clinical studies demonstrate that these metabolic improvements following bariatric surgery are accompanied by measurable gains in cognitive function, suggesting that restoration of metabolic homeostasis may positively influence brain health [143]. Emerging evidence further supports the concept that bariatric surgery alters gut–brain signaling and systemic metabolic pathways in ways that may reduce neural vulnerability to metabolic and inflammatory stressors [144]. Reviews of postoperative outcomes consistently report improvements in attention, memory, and executive function, reinforcing the potential cognitive benefits of surgical metabolic interventions [145].

Finally, normalization of cardiometabolic risk factors—including blood pressure, lipid levels, and body weight—appears critical for disrupting the pathological cardio–metabolic–brain axis. Therapeutic strategies that target insulin resistance as a central mechanism may therefore simultaneously mitigate cardiovascular burden and cognitive decline, highlighting insulin sensitivity as a unifying and modifiable therapeutic target across metabolic and brain health domains [146].

## 4. Discussion

Growing evidence suggests that insulin resistance (IR) contributes to cognitive decline not only in the context of overt metabolic disease but also during earlier, subclinical stages of metabolic dysfunction. Population-based analyses in non-diabetic aging cohorts demonstrate that reduced insulin sensitivity, estimated using the glucose disposal rate, is independently associated with poorer cognitive performance and accelerated cognitive aging, indicating that IR may precede clinically evident disturbances in glucose metabolism [60]. These findings support the concept that impaired insulin action represents an early systemic stressor capable of influencing brain function before the onset of type 2 diabetes.

Mechanistic insights from studies in individuals with established type 2 diabetes further reinforce this association, showing that chronic hyperinsulinemia, impaired glucose utilization, and low-grade inflammation act synergistically to disrupt neuronal energy homeostasis and synaptic integrity [133]. As metabolic dysregulation progresses, sustained exposure to oxidative stress and inflammatory mediators exacerbates neuronal vulnerability, thereby creating conditions conducive to cognitive impairment. Importantly, these systemic processes converge on insulin signaling pathways within the central nervous system, where insulin plays a pivotal role in maintaining neuronal viability and synaptic efficiency.

Within the brain, insulin signaling—particularly in the hippocampus—regulates neuroplasticity, synaptic remodeling, and long-term potentiation, processes that are essential for learning and memory. Disruption of insulin and IGF-1 signaling pathways has been shown to impair hippocampal neuroplasticity and reduce adaptive neuronal responses, thereby linking peripheral metabolic abnormalities to central cognitive dysfunction [143]. These molecular and cellular alterations are consistent with neuroimaging and neuropathological observations in metabolically compromised populations.

Longitudinal evidence further indicates that metabolic health is dynamically related to cognitive outcomes. Changes in metabolic syndrome status over time are accompanied by corresponding changes in cognitive performance, suggesting that metabolic risk factors exert cumulative but potentially reversible effects on brain function [144]. In this context, insulin resistance has emerged as a key determinant of a broader metabolic signature that predicts neurodegenerative risk, including biochemical patterns associated with Alzheimer’s disease pathology [76]. Such findings underscore the role of IR as a systemic biomarker linking metabolic dysregulation to progressive cognitive decline.

Neuroimaging studies provide additional evidence linking insulin resistance to altered brain network organization. In older adults with obesity, higher levels of IR are associated with disrupted functional connectivity, reduced network efficiency, and impaired integration of cognitive control and memory networks, suggesting that metabolic dysfunction extends beyond regional brain changes to affect large-scale neural systems [89].

Collectively, these findings align with the emerging concept of “Type 3 Diabetes,” which frames Alzheimer’s disease and related dementias as disorders partially driven by brain-specific insulin resistance [145]. Within this framework, insulin resistance functions as a unifying pathophysiological mechanism linking metabolic dysfunction to structural, functional, and molecular brain alterations. This integrative perspective reinforces the relevance of the cardio–metabolic–brain axis and highlights insulin resistance as a critical target for early intervention aimed at preventing both cardiovascular and neurodegenerative disease.

## 5. Conclusions

Insulin resistance emerges from this review as a central pathophysiological process linking metabolic disorders, cardiovascular disease, and progressive impairment of brain structure and function. Accumulating evidence indicates that insulin resistance, particularly in the context of obesity and type 2 diabetes, extends far beyond altered glucose metabolism and represents a systemic condition affecting vascular integrity, inflammatory balance, and neuronal energy homeostasis. The cardio–metabolic–brain axis provides a useful integrative framework that connects laboratory biomarkers of metabolic dysfunction with vascular injury and early markers of neurodegeneration. Importantly, both metabolic and imaging biomarkers highlight that adverse brain changes may develop long before overt cognitive symptoms become clinically apparent. These observations underscore the clinical relevance of early identification of insulin resistance and related metabolic abnormalities as part of cardiovascular and cognitive risk stratification. Interventions targeting insulin sensitivity—through lifestyle modification, pharmacotherapy, or metabolic surgery—may therefore offer a unique opportunity to simultaneously reduce cardiometabolic burden and preserve brain health. Future research should focus on longitudinal, multimodal studies integrating metabolic, vascular, and neural biomarkers within the same individuals to refine risk phenotyping and support personalized preventive strategies aimed at interrupting the cardio–metabolic–brain axis at its earliest stages.

## Figures and Tables

**Table 1:** Key characteristics of the included studies.

Authors	Title	Journal	Type of Article	Number of Participants
Tong H	Brain Insulin Signaling is Associated with Late-Life Cognitive Decline	Aging and Disease	Original research	1194
Zhou M	Blood Pressure Partially Mediated the Association of Insulin Resistance and Cerebral Small Vessel Disease: A Community-Based Study.	Am Heart Assoc	Original research	2752
Li R	Associations of Glucose Metabolism Status with Brain Macrostructure and Microstructure: Findings from the UK Biobank.	Journal of Clinical Endocrinology and Metabolism	Original research	29,251
Zhang Z	Insulin resistance assessed by estimated glucose disposal rate and risk of incident cardiovascular diseases among individuals without diabetes: findings from a nationwide, population based, prospective cohort study.	Cardiovasc Diabetol	Original research	5512
Yang XY	The association between triglyceride–glucose index and the recurrence of myocardial infarction in young patients with previous coronary heart disease.	Sci Rep.	Original research	1013
Yang W	The Metabolic Score for Insulin Resistance (METS-IR) Predicts Cardiovascular Disease and Its Subtypes in Patients with Hypertension and Obstructive Sleep Apnea.	Clin Epidemiol	Original research	2031
Landowska M	Is Insulin Resistance an Independent Predictor of Atherosclerosis?	J Clin Med.	Original research	178
Behiry EG	Evaluation of TG-HDL Ratio Instead of HOMA Ratio as Insulin Resistance Marker in Overweight and Children with Obesity	Endocr Metab Immune Disord Drug Targets	Original research	90
Cui DY	Associations between non-insulin-based insulin resistance indices and heart failure prevalence in overweight/obesity adults without diabetes mellitus: evidence from the NHANES 2001–2018	Lipids Health Dis	Original research	13,473
Bruehl H	Obese Adolescents with Type 2 Diabetes Mellitus Have Hippocampal and Frontal Lobe Volume Reductions	Neurosci Med.	Original research	36
Willette AA	Association of insulin resistance with cerebral glucose uptake in late middle-aged adults at risk for Alzheimer disease	JAMA Neurol.	Original research	150
Cicarelli DD	Comparison of C-reactive protein and serum amyloid A protein in septic shock patients	Mediators Inflamm.	Original research	29
Musen G	Resting-state brain functional connectivity is altered in type 2 diabetes	Diabetes.	Original research	21
Cui Y	Aberrant functional connectivity of default-mode network in type 2 diabetes patients	Eur Radiol.	Original research	84
Kullmann S	The obese brain: Association of body mass index and insulin sensitivity with resting state network functional connectivity	Hum Brain Mapp.	Original research	23
Li J	Network efficiency of functional brain connectomes altered in type 2 diabetes patients with and without mild cognitive impairment	Diabetology and Metabolic Syndrome.	Original research	54
McIntyre CC	Insulin resistance, cognition, and functional brain network topology in older adults with obesity	Sci Rep.	Original research	180
Laws SM	Insulin resistance is associated with reductions in specific cognitive domains and increases in CSF tau in cognitively normal adults.	Sci Rep.	Original research	1264
Xiu M	Glucose metabolism, hippocampal subfields and cognition in first-episode and never-treated schizophrenia	International Journal of Clinical and Health Psychology	Original research	29
Willette AA	Association of insulin resistance with cerebral glucose uptake in late middle-aged adults at risk for Alzheimer’s disease	JAMA Neurology	Original research	150
van Gils V	Associations Between Glucose Metabolism Measures and Amyloid-β and Tau Load on PET 14 Years Later: Findings From the Framingham Heart Study	Diabetes Care	Original research	288
Weinstein G	Glucose indices are associated with cognitive and structural brain measures in young adults	Neurology	Original research	6553
Bove RM	Association between adiposity and cognitive function in young men: hormonal mechanisms	Obesity	Cross-sectional study	53
Frazier DT	Relationship between Insulin-Resistance Processing Speed and Specific Executive Function Profiles in Neurologically Intact Older Adults	Journal of the International Neuropsychological Society	Original research	119
Weinstein G	Non-alcoholic fatty liver disease, liver fibrosis score and cognitive function in middle-aged adults: The Framingham Study.	Liver International	Original research	1287
Shima A	Glucose metabolism and smaller hippocampal volume in elderly people with normal cognitive function	npj Aging	Original research	11,957
Geroldi C	Insulin Resistance in Cognitive Impairment: The InCHIANTI Study.	Arch Neurol.	Original research	523
Willmann C	Insulin sensitivity predicts cognitive decline in individuals with prediabetes.	BMJ Open Diabetes Res Care	Original research	160
Toppala S	Midlife Insulin Resistance as a Predictor for Late-Life Cognitive Function and Cerebrovascular Lesions	Journal of Alzheimer’s Disease	Original research	6062
Willette AA	Insulin resistance, brain atrophy, and cognitive performance in late middle-aged adults.	Diabetes Care	Original research	372
Ni W	Altered brain functional network connectivity and topology in type 2 diabetes mellitus.	Front Neurosci.	Original research	152
Zhao H	Insulin Resistance Is a Risk Factor for Mild Cognitive Impairment in Elderly Adults with T2DM	Open Life Sciences	Original research	78
McIntyre CC	Insulin Homeostasis Mediates the Relationship Between Cardiorespiratory Fitness and Cognitive Speed in Aging Adults	Journal of Alzheimer’s Disease	Original research	1131
Ennis GE	Insulin resistance is related to cognitive decline but not change in CSF biomarkers of Alzheimer’s disease in non-demented adults	Alzheimer’s and Dementia: Diagnosis, Assessment and Disease Monitoring	Original research	1384
Martín-Saladich Q	Brain [^18^F]FDG uptake patterns in type 2 diabetes: new phenotypes relating to biomarkers of cognitive impairment	Brain Communications	Original research	51
Vreeken D	Factors Associated With Cognitive Improvement After Bariatric Surgery Among Patients With Severe Obesity in the Netherlands	JAMA network open	Original research	156
Custers E	Sustained Improvement of Cognition, Mood and Plasma Markers Three Years After Metabolic Bariatric Surgery. The BARICO Study	Obesity Surgery	Original research	107
Wang B	Evaluating the link between insulin resistance and cognitive impairment using estimated glucose disposal rate in a non-diabetic aging population: results from the CHARLS	Frontiers in Medicine	Original research	5178
Frentz I	Metabolic Syndrome Status Changes and Cognitive Functioning: Insights from the Lifelines Cohort Study	Journal of Prevention of Alzheimer’s Disease	Original research	14,609

## Data Availability

No datasets were generated or analysed during the current study.
